# Chemical disaggregation of alpha-synuclein fibrils as a therapy for synucleinopathies

**DOI:** 10.1073/pnas.2300965120

**Published:** 2023-03-08

**Authors:** Shenjie Wu, Nancy C. Hernandez Villegas, Randy Schekman

**Affiliations:** ^a^Department of Molecular and Cell Biology, University of California, Berkeley, CA 94720; ^b^HHMI, University of California, Berkeley, CA 94720; ^c^Helen Wills Neuroscience Institute, University of California, Berkeley, CA 94720

Protein aggregate formed by alpha-synuclein is the hallmark of a series of neurodegenerative disorders known as synucleinopathies (also known as Lewy body diseases), including Parkinson’s disease (PD), dementia with Lewy bodies, and multiple system atrophy (MSA) (1). Globally PD alone affects the lives of more than 10 million people. In synucleinopathies, soluble, monomeric alpha-synuclein aggregates into fibrillar structures. The fibrillar alpha-synucleins, together with other protein aggregates, lipid, and damaged organelles constitute the insoluble inclusions, Lewy Bodies, seen in postmortem brain tissue from PD patients. The presence of both alpha-synuclein oligomers and fibrils has been suggested to contribute to the cytotoxicity in the pathogenesis of synucleinopathies (2). Like many neurodegenerative diseases, the current treatment for PD and other synucleinopathies is palliative, aiming to control the symptoms with regrettably little impact on the progression of the disease. Alpha-synuclein fibrils represent an obvious therapeutic target given the possible role of these aggregates in the etiology of synucleinopathies. In this issue of the Proceedings, Murray et al. (3) report a new class of compounds with the ability to disaggregate alpha-synuclein fibrils both in vitro and in vivo.

**Fig. 1.**
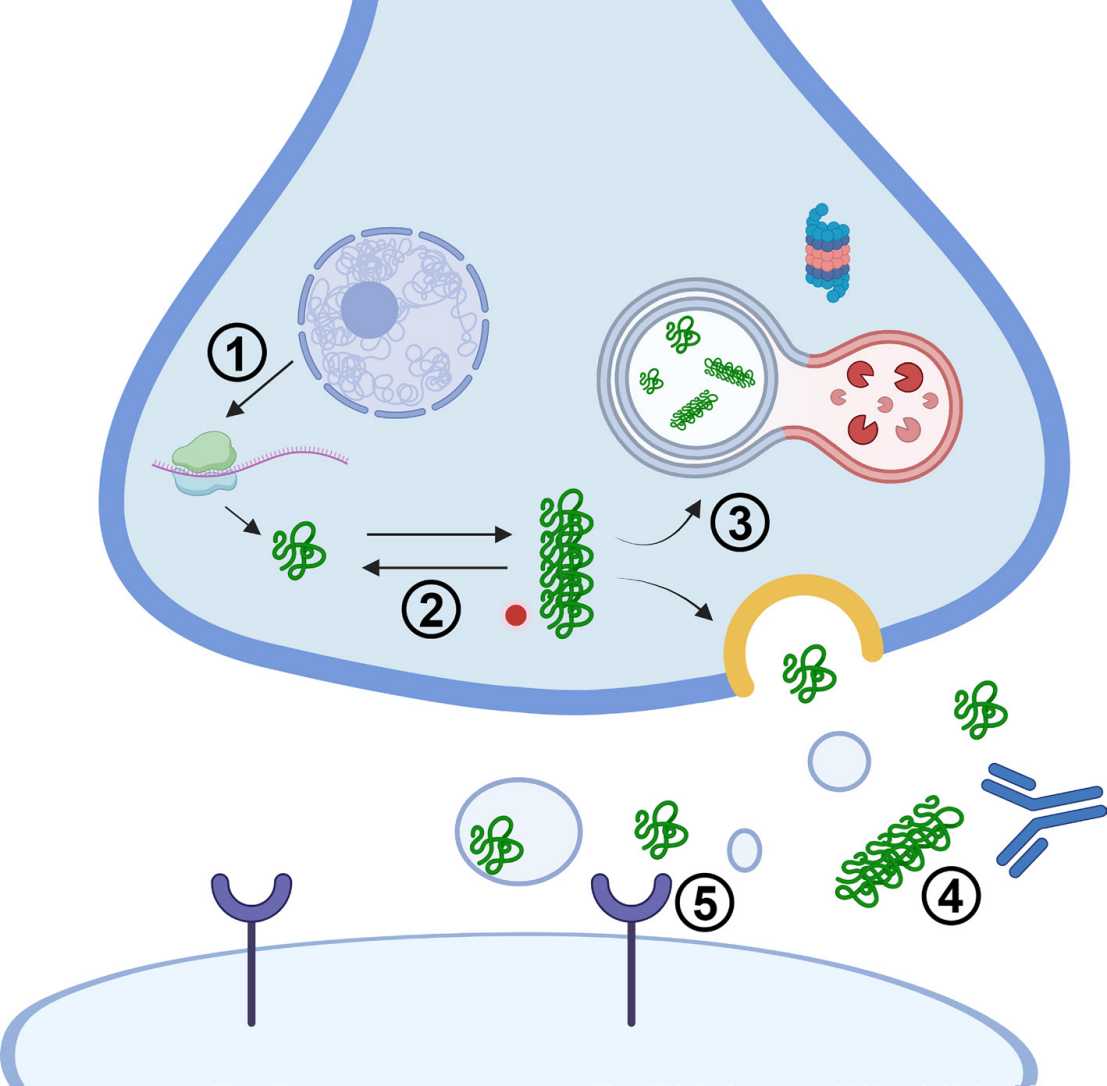
Different approaches of therapeutic intervention to mitigate alpha-synuclein aggregate toxicity. (1) Reduction in alpha-synuclein expression by gene therapy, e.g., RNA interference, antisense oligonucleotide (ASO). (2) Inhibition of monomeric alpha-synuclein polymerization or dissolving oligomers and fibrils with peptides or small molecules. (3) Target degradation of alpha-synuclein fibrils by proteasome or autophagy/lysosome machineries. (4) Neutralization of extracellular alpha-synuclein fibrils with antibodies/nanobodies. (5) Block putative alpha-synuclein fibril receptor to prevent internalization and spread of pathology. Mechanistic studies of all these pathways may shed light on innovative therapeutic approaches in the future.

Much effort has been expended to clear alpha-synuclein aggregates at different stages of its life cycle (Fig. 1), but, as yet, no drug or immune therapy has shown promise in modifying disease progression. In addition to aggregation per se, alpha-synuclein aggregates can seed endogenous soluble counterparts to form new aggregates and possibly spread between cells (4). One of the approaches used to stop the aggregation and spread of alpha-synuclein was to knockout alpha-synuclein or to delete its nonamyloid component region (5). Although animal experiments showed promising results, the long-term effects of alpha-synuclein deletions must be balanced against the possibly important roles of this protein in synaptic plasticity and neurotransmitter release (6), and other functions, e.g., messenger RNA stability (7). Short-term ablation of gene expression with the use of antisense oligonucleotide (ASO) also produced a reduction in the levels of endogenous alpha-synuclein in the brain of mouse, rat, and nonhuman primate models with a corresponding decrease in aggregation and spread of pathology (8). Antibody-based immunotherapy has been attempted with little success to prevent the uptake and subsequent cell-to-cell transmission of alpha-synuclein fibrils (9). Other studies have explored drugs and peptides that may target pathways to enhance the clearance of alpha-synuclein in the brain. In one recent study, irisin, an exercise-induced hormone, was found to decrease the internalization and boost the lysosomal degradation capacity of alpha-synuclein fibrils (10). Wang et al. (11) reported engineered peptides that can cross the blood–brain barrier (BBB) and the plasma membrane, bind specifically to the monomeric form of alpha-synuclein, and direct it to the lysosome or proteasome for degradation.

The study by Murray et al. on alpha-synuclein fibrils and related work on tau fibrils highlights the rapid development of aggregate targeting approaches in the battle against neurodegenerative diseases.

A more direct therapeutic strategy has been to use peptides and small molecules that prevent or reverse the aggregation of proteins implicated in neurodegenerative diseases such as Alzheimer’s disease (AD) and PD. The challenge for any neurospecific drug has been to create molecules of great specificity and selectivity that can pass the BBB. Amyloidogenic small fibril fragments generated by inefficient disaggregation may exacerbate rather than alleviate pathology. Recent structures determined by cryoelectron microscopy reveal heterogeneity among alpha-synuclein fibrils from different sources of isolated recombinant protein and human origin, with changes seen even as the disease progresses (12). The challenge is made greater by the need to target multiple possible species while avoiding toxic interactions with normal processes of assembly of other protein oligomers or filaments.

Murray et al. (3) began their effort by building upon previous structural studies of tau fibrils bound by a natural disaggregation compound derived from green tea, flavonoid epigallocatechin gallate (13). Within a series of potential disaggregation compounds designed with rules that optimize for brain penetrant properties, two molecules termed CNS-11 and its analog CNS-11g showed robust disaggregation activity toward preformed alpha-synuclein fibrils in vitro. Using a biosensor aggregation reporting system and an in vivo test of toxicity (14), the authors demonstrated that disaggregated alpha-synuclein fibrils lose their ability to template endogenous soluble alpha-synuclein at concentrations that are tolerated by cells and *Caenorhabditis elegans* nematodes. The inhibition of seeding by these compounds could mean that newly solubilized alpha-synuclein monomer or oligomer has reduced amyloidogenic properties. This is important because small fibrillar fragments generated by incomplete disaggregation could serve as even more potent seeds (15).

Extending their analysis of preformed alpha-synuclein fibrils, Murray et al. (3) report that CNS-11 and CNS-11g also efficiently disaggregated and prevent seeding of alpha-synuclein fibrils isolated from brain tissue from an MSA patient. Molecular dynamic simulations suggest that CNS-11 and CNS-11g bind the N terminus of alpha-synuclein, which forms the core in many reported fibril structures derived from different sources (16, 17). Thus, the same compounds may also help dissolving alpha-synuclein fibrils from other synucleinopathies. In an in vivo model using *C. elegans*, CNS-11 and CNS-11g were found to decrease the number of puncta formed by alpha-synuclein aggregates. CNS-11 and CNS-11g introduced by vein injection into mice were found in both blood plasma and brain samples, suggesting these compounds, as predicted, had at least some brain penetrance. Although the concentrations achieved from a single administration appear not to be at a level that could elicit fibril disaggregation properties in vivo, this represents but the first step in a long process of dosing and drug optimization.

The hard work of producing and testing analogs that improve the specificity, reduce toxicity and other vital parameters of drug discovery lies ahead. The study by Murray et al. (3) on alpha-synuclein fibrils and related work on tau fibrils highlights the rapid development of aggregate targeting approaches in the battle against neurodegenerative diseases. Other targets and approaches must be pursued in the quest to break the inexorable advance of disease and death attributable to AD and PD and related diseases.
